# CVID Enteropathy Associated With Chronic Norovirus Infection: Background, Clinical Features, and Therapeutic Aspects

**DOI:** 10.1002/rmv.70081

**Published:** 2025-11-13

**Authors:** Györgyi Műzes, Ferenc Sipos

**Affiliations:** ^1^ Immunology Division Department of Hematology and Internal Medicine Semmelweis University Budapest Hungary

**Keywords:** chronic diarrhoea, common variable immunodeficiency, CVID enteropathy, norovirus, oral immunoglobulin

## Abstract

Common variable immunodeficiency (CVID) is the most prevalent symptomatic primary immunodeficiency, characterised by impaired antibody production, immune dysregulation, and a broad spectrum of clinical manifestations. Gastrointestinal involvement is frequent, affecting up to 20% of patients and significantly contributing to morbidity and mortality. Among infectious triggers, norovirus plays a particularly important role, as persistent infection may drive chronic inflammation and contribute to the development of CVID‐associated enteropathy, a severe non‐infectious complication marked by chronic diarrhoea, malabsorption, and weight loss. The pathogenesis is multifactorial, involving impaired humoural immunity, absent mucosal IgA, and aberrant T‐ and B‐cell interactions, resulting in defective viral clearance and sustained mucosal injury. Although viral eradication has been shown to induce clinical and histological improvement, no standardized therapeutic strategy currently exists. Intravenous or subcutaneous immunoglobulin replacement fails to adequately protect against gastrointestinal infections, and off‐label antivirals such as ribavirin, nitazoxanide, or interferon alpha have yielded inconsistent results. Oral administered immunoglobulin preparations have shown variable efficacy in case reports, reflecting differences in viral genotypes, host susceptibility, and donor antibody repertoires. In this review, we summarise current knowledge on the epidemiology, pathogenesis, clinical features, and diagnostic considerations of CVID‐associated enteropathy linked to chronic norovirus infection, with a special focus on therapeutic aspects. We also present our experience with a patient successfully treated with immunoglobulin therapy administered via nasogastric tube, leading to clinical remission, nutritional recovery, and viral clearance. Recognising norovirus as a key etiological factor in CVID enteropathy emphasises the need to conduct systematic studies and evidence‐based therapeutic approaches.

## 1 Introduction

Primary immunodeficiency disorders (PID), also known as inborn errors of immunity, remain a neglected field of medicine, despite the significant progress made in it over the past few decades [1, 2]. Common variable immunodeficiency (CVID) is a primary immunodeficiency disorder that is relatively prevalent [3]. It is distinguished by impaired antibody production, often accompanied by T‐cell abnormalities, which results in an increased susceptibility to infections, particularly those of the respiratory, urinary, and gastrointestinal tracts [3]. It is most commonly observed during adolescence or early adulthood, but it can manifest at any age [4, 5]. CVID is a disorder that is heterogeneous in nature, characterised by a complex genetic background and variable clinical manifestations. Although the majority of cases are sporadic, a small number of them exhibit familial clustering [6, 7]. In certain patients, mutations in genes that are involved in B cell development and function, including TNFRSF13B (encoding TACI), ICOS, CD19, and others, have been identified [7]. Nevertheless, no single genetic defect is responsible for all cases.

Like any other PID, CVID impairs the three main functions of the immune system. The failure to protect against pathogens leads to the development of various infections. Dysregulation of anti‐tumour immunity also increases the risk of solid and haematological malignancies. The disruption of self‐tolerance promotes the development of autoimmune phenomena. To mitigate the frequency and severity of infections and prevent long‐term complications, early diagnosis and intravenous or subcutaneous immunoglobulin replacement (IVIG or SCIG) therapy are indispensable [3].

Gastrointestinal (GI) disease is reported in over 20% of the CVID populations [8, 9]. In comparison to controls of the same age and sex, this complication results in a substantial increase in mortality [4]. Non‐infectious conditions (e.g., atrophic gastritis, pernicious anaemia, nodular lymphoid hyperplasia, CVID enteropathy, coeliac‐like disease, inflammatory bowel‐like disease, autoimmune hepatitis, nodular regenerative hyperplasia, MALT‐lymphomas, and GI tract cancers) can resemble well‐established gastrointestinal disorders, but they exhibit distinct histopathological findings that necessitate specific therapies [10, 11]. It has been reported that the efficacy of IVIG substitution therapy in treating GI infections is reduced by its lower capacity to bind intestinal microbiota in CVID patients with infection‐related GI conditions [12]. CVID is frequently associated with immunoglobulin A (IgA) deficiency, which elevates the likelihood of gastrointestinal infections [13]. In patients with CVID, GI infections may respond atypically to standard treatments and manifest protracted clinical courses [10].

*Giardia* sp., norovirus, *Salmonella* spp., and *Campylobacter* spp. are predominant etiological agents of infectious gastroenteritis in individuals with CVID [14, 15]. Symptoms are typically nonspecific and vary from self‐limiting to chronic or refractory gastrointestinal discomfort, diarrhoea, and weight loss. A favourable correlation exists between *Giardia*, *Salmonella*, and *Campylobacter* infections in CVID patients exhibiting undetectable serum IgA levels, which is expected considering the recognized function of IgA in mucosal immunity [13, 16]. Norovirus and cytomegalovirus infections seldom possess clinical relevance in immunocompetent individuals; nonetheless, their sequelae might be severe in cases of CVID [10].

Norovirus gastroenteritis is a predominant cause of gastroenteritis globally [17]. Its prevalence has been determined to be 7% among CVID patients [15]. Norovirus impacts 19% of the global population; however, it has been detected in up to 100% of individuals with CVID enteropathy [17, 18]. Chronic norovirus infections have been recognized as a cause of CVID enteropathy [19]. Consequently, individuals with CVID and concomitant norovirus infections must be actively followed for symptom remission, and alternate treatments should be contemplated if infections persist.

However, currently, there is no established conventional therapy for chronic norovirus infection resulting in CVID enteropathy. In the absence of systematic comprehensive studies, we must depend solely on case reports. We recently diagnosed and successfully treated a patient with CVID‐associated enteropathy related to chronic norovirus infection, which constitutes the central focus of this review. Our objective is to provide a concise overview of this distinct form of primary immunodeficiency, drawing on the current—albeit still incomplete—body of knowledge.

## 2 Epidemiology

### 2.1 CVID

The prevalence of CVID is estimated to be between 1 in 25,000 and 1 in 50,000 individuals, with a greater frequency in Northern Europe [5, 6, 9, 13, 20, 21]. It is predominantly diagnosed post‐puberty, with the majority of cases occurring between the ages of 20 and 45 years [4, 9, 13]. It exhibits no preference for race or gender.

### 2.2 Norovirus Gastroenteritis

Norovirus causes approx. 685 million gastroenteritis cases, 150,000 adult fatalities, and 50,000 child deaths annually [22]. Although lower‐income countries face the largest disease burden, norovirus outbreaks occur worldwide [23, 24]. Mild disease patients may not seek treatment; hence, the number of cases may surpass the estimate. Food‐borne illness was the most common cause of norovirus outbreaks. Ingesting food contaminated during production or by food service workers during preparation might cause food‐related transmission. Raw fruits, vegetables, oysters, and seafood are high‐risk norovirus diets. Norovirus can affect anyone, although the elderly and immunocompromised are more at risk. Some studies reveal a 7% 30‐day death rate for community‐acquired norovirus in older persons [25, 26]. Neonates also develop more significant problems, including necrotising enterocolitis. Young children have the most norovirus cases [26]. The annual incidence of norovirus in children under five is estimated at 21,400 per 100,000 [22]. Norovirus infections are also more common in underdeveloped countries. It has been calculated that norovirus causes 17% of developing country gastroenteritis [27]. Immunocompromised patients are also affected by norovirus. Patients with impaired immune systems are more likely to contract norovirus, develop problems, and shed virus asymptomatically [28].

### 2.3 CVID Enteropathy

According to a hospital‐based study, the prevalence of gastrointestinal enteropathy in patients with CVID is estimated at 2%–4% [29, 30]. In a larger cohort of 728 CVID patients, 15.6% were found to have some form of CVID‐associated enteropathy [31]. Furthermore, data from a retrospective database analysis (USIDNET registry) reported gastrointestinal disorders, including chronic diarrhoea and colitis, in 20% of all CVID patients [32].

## 3 Pathogenesis

The genetic susceptibility of patients with CVID to norovirus infections is currently not well defined and is linked to a specific genetic factor. However, there is a correlation between several key immunological and genetic factors.

### 3.1 Norovirus

Noroviruses are classified under the Caliciviridae family and are categorised into multiple genogroups (GI to GVIII) according to their genetic composition [33]. Genogroups I, II, and IV are recognized as human pathogens, with genogroup II (GII) being the most widespread. Each genogroup comprises many genotypes or subtypes, which may vary in their genetic composition and antigenic characteristics [34]. Since the initial incidence of Norovirus in a school in Sapporo, Hokkaido, Japan, in 1996, the epidemiology of noroviruses has evolved [35, 36, 37, 38]. Reports indicate that the regular introduction of novel genotypes and lineages has led to the replacement of endemic viruses across all genotypes. The introduction of novel genotypes into a serologically naïve population may lead to unusual outbreaks and is linked to more severe manifestations of norovirus [39].

The pathophysiology of norovirus infection entails a complicated interaction among viral entrance, replication, and the host's immunological response. The virus predominantly infects the gastrointestinal system, specifically targeting intestinal epithelial cells as well as immune cells such as macrophages and dendritic cells [40, 41]. The virus predominantly affects the small intestine, impairing the intestinal lining and hindering nutrient absorption [41].

Noroviruses are predominantly spread through the faecal–oral route, primarily via the consumption of contaminated food or water or through contact with contaminated surfaces. The virus commences infection by attaching to histo‐blood group antigens (HBGAs) on host cells, which act as receptors, with various norovirus strains demonstrating unique HBGA binding specificities [40, 41, 42]. Subsequent to attachment, the virus infiltrates host cells via endocytosis [41]. Noroviruses infect intestinal epithelial cells as well as immune cells, such as macrophages and dendritic cells [42]. While the exact methods of viral replication in human cells are not fully elucidated, evidence suggests that norovirus infection compromises the integrity of the intestinal mucosa by destroying microvilli and modifying tight junctions [40]. These structural alterations hinder nutritional absorption—impacting, among others, fat and d‐xylose—and facilitate the onset of diarrhoea [40].

### 3.2 Genetic Background

Galactoside 2‐alpha‐L‐fucosyltransferase 2 is an enzyme that in humans is encoded by the FUT2 (fucosyltransferase 2) gene [43]. The FUT2 gene determines whether ABH blood group antigens are expressed on mucosal surfaces, classifying individuals as either ‘secretors’ or ‘non‐secretors’ [44]. Approximately 20% of the Caucasian population carry a non‐secretor status due to a loss‐of‐function mutation in FUT2, which confers substantial protection against the GII.4 strain of norovirus, the most common causative agent of infection [45, 46, 47]. Meta‐analyses have shown that ‘secretor’ individuals may have up to a tenfold higher risk of GII.4 infection and about a twofold higher risk for non‐GII.4 strains compared with non‐secretors [47].

In addition to GII.4, the GII.6 genotype has recently constituted a significant number of norovirus cases, sometimes ranking as the second most prevalent. From 2005 to 2016, the annual prevalence of GII.6 in Europe varied from 2.0% to 10.3% of strains reported to the NoroNet network [48]. Furthermore, GII.6 strains constituted an average of 5.4% of norovirus outbreaks in the USA reported to CaliciNet from 2013 to 2016, peaking at 10.3% in 2014–2015, thereby ranking as the second most prevalent genotype associated with outbreaks that year [49]. Concerning the GII.6 genotype, HBGA characterisation indicated a robust link between secretor status and norovirus‐related symptoms. However, no correlation was found between norovirus infection and ABO blood types [50].

It is important to note that these associations reflect general mechanisms of host susceptibility to norovirus and are not specific to the pathophysiology of CVID.

At the same time, certain monogenic causes of the CVID phenotype (e.g., NFKB1, NFKB2, TNFSF12/TWEAK/, TNFRSF13B/TACI/, or IKZF1/IKAROS/) have been reported to cause severe viral infections individually [51, 52, 53, 54, 55]. Some of these genes are responsible for encoding pattern recognition receptors (PRR) or proteins involved in natural killer cell (NK) and cytotoxic T‐lymphocyte (CTL) cytotoxicity (TWEAK). Dysfunction of other genes can lead to the production of anti‐cytokine antibodies or to the malfunctioning of cytotoxic mechanisms [54, 56].

### 3.3 Host Gut Microbiota

In addition to genetic susceptibility variables, the nature of the host gut microbiota significantly influences the modulation of norovirus infection [46]. Some bacteria that live in the intestines, like *Enterobacter cloacae*, *Escherichia coli*, and *Helicobacter pylori*, have carbohydrates on their surface that are similar to HBGA. These carbohydrates can stick to norovirus particles, making it easier for them to adhere to host cells and making the virus more infectious [57, 58, 59]. Experimental studies have shown that norovirus can only infect B cells when HBGA‐coated bacteria are present. This suggests that bacterial adhesion helps the virus get into cells [57]. Furthermore, bacteria that express these HBGA‐like molecules can shield norovirus particles from heat inactivation, hence facilitating environmental stability and transmission [60]. On the other hand, some types of bacteria, including *Lactobacillus*, may have antiviral effects by boosting the immune system or competing for viral binding sites [61, 62]. Consequently, the gut microbiota can either facilitate or inhibit norovirus infection, contingent upon the bacterial species involved and their interactions with the host's immunological and glycobiological milieu [62, 63].

### 3.4 Immunological Factors

In CVID, humoural immunity is profoundly impaired, particularly due to the lack of IgA production, which hampers the effective clearance of viruses, including norovirus [64]. However, cellular immunity can also be impacted: 40% of cases exhibited reduced T‐cell responses to mitogens, and 20% of cases had low CD4 counts [4]. The function of innate immunity in CVID, which includes the production of anti‐viral cytokines, particularly type I (IFN‐α and IFN‐β) and type III interferons (IFN‐γ), and viral recognition by PRRs, is generally unaffected [65].

The adaptive antiviral immune response is mostly mediated by the identification and elimination of host cells by CTLs and NKs, via MHC Class I molecules and particular NK receptors, respectively [64, 66]. CTLs are traditionally classified as CD8+, although it is increasingly recognized that during acute and chronic viral infections, CD4+ T‐cell subsets with cytotoxic capabilities are produced and may even supplant CD8+ T‐cells in chronic viral infections [66]. CD4+ T‐cells are traditionally linked to *T* helper (Th) cell subsets; however, naïve CD4+ T‐cells can develop into other effector cell subsets based on activation from antigen presentation cells (APC) and additional variables [66]. Th1 T‐cells are generated during viral infections and stimulate the synthesis of antiviral cytokines, enhancing cytotoxicity [66].

The efficacy of vaccines in preventing virus infections illustrates the antiviral function of humoural immunity, as it is facilitated by the rapid generation of neutralising antibodies against the circulating virus. Furthermore, antibody‐dependent cellular cytotoxicity (ADCC) is a mechanism by which NK cells and CTLs can identify and eliminate virus‐infected host cells [64, 66]. B‐cells are essential for antiviral responses due to their ability to produce antibodies, modulate the immune response by secreting cytokines, and serve as APCs to T‐cells through the use of MHC class II molecules. As a consequence, B‐cell dysfunction leads to chronic and recurrent viral infections, as observed in CVID or X‐linked agammaglobulinemia [13, 67].

**FIGURE 1:**
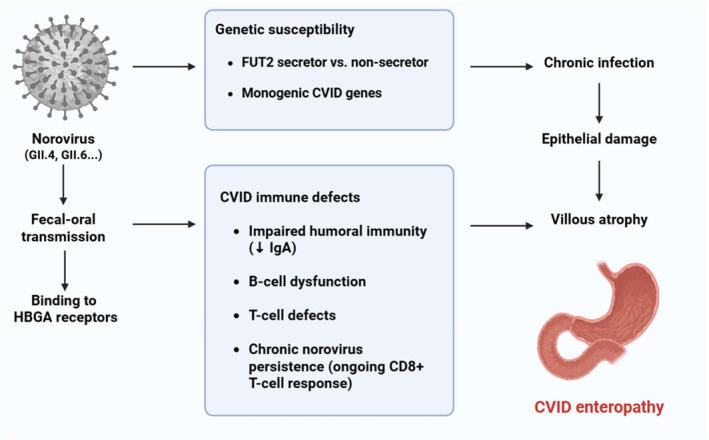
Presumed pathogenesis of CVID‐associated enteropathy complicated by chronic norovirus infection. The diagram illustrates the pathogenesis of CVID enteropathy within the context of chronic norovirus infection. Norovirus enters via the faecal–oral route, binds to histo‐blood group antigens (HBGAs), and infects intestinal epithelial and immune cells. Host genetic susceptibility (e.g., FUT2 secretor status, monogenic CVID genes) and CVID‐related immune defects (impaired humoural immunity, B‐cell dysfunction, T‐cell abnormalities) impair viral clearance, resulting in persistent infection. Chronic CD8+ T‐cell–mediated epithelial damage leads to villous atrophy and CVID enteropathy. The figure was created partly with BioRender (biorender.com).

CVID patients may experience chronic norovirus infections lasting for months or even years [68]. In some cases, persistent norovirus infection can trigger or exacerbate CVID‐associated enteropathy [69]. The production of neutralising antibodies, the interaction between T‐cells and B‐cells, and the release of mediators from CD4+ T‐lymphocytes are all impaired in CVID due to the compromise of B‐cell differentiation to plasmatic cells [68, 69]. Consequently, the clearance of norovirus is disrupted, and a persistent and uncontrolled CD8+ T‐cell response results in epithelial injury, which ultimately leads to the typical villous mucosal atrophy [69]. Research has shown that the etiological function of norovirus in CVID enteropathy can be supported by the symptomatic and histological improvement that can result from viral clearance, such as with ribavirin [18, 19]. Figure 1 illustrates the presumed pathomechanism of CVID enteropathy complicated by chronic norovirus infection.

## 4 Clinical Aspects

CVID‐associated enteropathy is defined by chronic diarrhoea and malabsorptive symptoms in the absence of infectious, autoimmune, or gluten‐sensitive causes [31, 70, 71]. Histological hallmarks include villous atrophy, intraepithelial lymphocytosis, lymphoid hyperplasia, and duodenitis, with the definition increasingly incorporating clinical parameters such as unexplained weight loss and hypoalbuminemia [72]. Overall, it remains a diagnosis of exclusion supported by characteristic but heterogeneous histopathological findings [73, 74, 75].

### 4.1 Disease Manifestation

#### 4.1.1 Clinical Symptoms

After a short incubation period of 12–48 h, norovirus symptoms generally manifest as moderate gastrointestinal disturbances, including nausea, vomiting, diarrhoea, and abdominal pain, frequently accompanied by fever, chills, headache, general malaise, and myalgia [76]. In healthy, immunocompetent individuals, norovirus symptoms typically resolve within 2–3 days; however, in immunocompromised patients or those with other underlying conditions, a severe and persistent sickness may ensue [76, 77].

In CVID‐associated enteropathy, chronic watery diarrhoea represents the most frequent GI manifestation [31, 78]. Furthermore, reports indicate that up to 38% of patients experience dyspeptic symptoms and abdominal pain [78]. Fever may occur in approximately 40% of cases as a systemic feature. Patients often exhibit a reduced body mass index (BMI), in some instances falling below 18 kg/m^2^. Malabsorption is a common finding, frequently accompanied by nutritional deficiencies such as anaemia, hypoalbuminemia, hypokalemia, hypomagnesaemia, and/or hypocalcemia [31, 78, 79].

Given the underlying immune dysregulation in CVID, a broad spectrum of concomitant autoimmune disorders may also occur, including autoimmune haemolytic anaemia, immune thrombocytopaenia, lymphocytic thyroiditis, primary biliary cholangitis, vitiligo, or psoriasis [80, 81, 82, 83, 84].

#### 4.1.2 Histological Alterations

Immunopathology of the stomach and duodenum has been documented in CVID inside the upper gastrointestinal system [74, 85]. A significant number of patients are diagnosed with chronic erythematous, follicular, atrophic, or ulcerative gastritis. It is important to remember that in the progression of chronic gastric inflammatory mucosal processes, *Helicobacter pylori* is not the sole factor; immunological dysregulation also significantly contributes. In adult patients with CVID, atrophic gastritis and intestinal metaplasia correlate with a heightened incidence of gastric malignancies, including carcinoma and lymphoma [74, 85].

In CVID‐associated enteropathy, characteristic histopathological alterations are typically observed [31, 86]. These include a marked reduction or even complete absence of plasma cells within both the lamina propria and the deep crypts, accompanied by pronounced lymphocytic infiltration in the crypt regions. The number of intraepithelial lymphocytes is usually increased, and the development of lymphoid follicles (lymphoid hyperplasia) can often be detected. Duodenal and small intestinal villous atrophy, prominent apoptosis, granulomas, and crypt distortion are also a common feature [31, 74, 86, 87, 88]. In specific instances, histological changes resembling graft‐versus‐host disease may be observed [74]. It is suggested that patients with severe symptoms be categorised as having severe CVID enteropathy, while those with intermittent or chronic diarrhoea, absent weight loss, malnutrition, or significant gastrointestinal loss should be classified as having non‐severe CVID enteropathy [74].

In CVID, the colon exhibits histological characteristics such as a paucity or absence of plasma cells, an elevation in crypt apoptotic bodies, intraepithelial lymphocytosis, and sometimes pronounced inflammation. Loss of goblet cells is infrequently observed in small bowel and/or colon specimens [89].

#### 4.1.3 Laboratory Findings

In CVID‐associated enteropathy, laboratory abnormalities typically reflect both the underlying immunological defect and the secondary consequences of chronic intestinal inflammation and malabsorption.

The characteristic immunological findings include marked hypogammaglobulinemia, with a profound reduction in serum IgG levels and frequently decreased IgA and/or IgM concentrations [90, 91]. Patients generally exhibit impaired specific antibody responses, such as absent or diminished responses to vaccination. Alterations in B‐cell subsets are also common, most notably a reduction in switched memory B cells, occasionally accompanied by an expansion of CD21^low^ B cells. T‐cell abnormalities may also be observed, including reduced CD4+ T‐cell counts, an inverted CD4:CD8 ratio, and impaired proliferative capacity [90, 91].

The gastrointestinal and malabsorptive features are characterised by hypoalbuminemia, often due to protein‐losing enteropathy, together with multiple nutritional deficiencies [8, 32]. These may include reduced levels of fat‐soluble vitamins (A, D, E, and K), vitamin B12, and folate, as well as iron deficiency anaemia, which may coexist with anaemia of chronic disease. Electrolyte disturbances, such as hypocalcemia and hypomagnesaemia, can develop in the setting of severe malabsorption, while hypocholesterolemia and low triglyceride levels may also be present [8, 32, 92].

Inflammatory and disease‐mimicking laboratory features further complicate the clinical picture. Some patients demonstrate elevated inflammatory markers, including ESR and CRP, although these are not consistently raised [86]. Importantly, despite the frequent presence of villous atrophy on intestinal biopsy, coeliac‐specific serology (anti‐tTG, EMA, and DGP antibodies) is typically negative, which helps to distinguish CVID enteropathy from true coeliac disease [90, 91]. Stool analyses may reveal increased faecal *α*‐1 antitrypsin consistent with protein loss, steatorrhoea, and elevated faecal calprotectin levels [73, 93, 94].

Malabsorption due to CVID enteropathy, chronic inflammation stemming from immunological dysregulation linked to CVID, and potentially related autoimmune endocrinopathies lead to distinctive signs of osteoporosis; nevertheless, growth hormone insufficiency or primary hypoparathyroidism is infrequently observed [95, 96, 97, 98].

### 4.2 Complications

Chronic norovirus‐associated enteropathy in CVID is frequently complicated by severe nutritional and metabolic consequences, including protein–energy malnutrition, multiple micronutrient deficiencies, electrolyte disturbances, cachexia, and osteoporosis [94, 95, 96]. Gastrointestinal sequelae encompass chronic diarrhoea, small intestinal bacterial overgrowth, mucosal ulcerations, and nodular lymphoid hyperplasia, while the risk of gastrointestinal malignancies, particularly gastric carcinoma and lymphoma, is significantly increased [11, 94, 99]. Systemic complications include refractory malabsorption, various forms of anaemia, and thromboembolic events related to hypoalbuminemia and chronic inflammation [71, 100, 101]. Collectively, these manifestations contribute to substantial morbidity and reduced quality of life, which points to enteropathy as one of the most serious non‐infectious complications of CVID.

## 5 Diagnostic Considerations

### 5.1 CVID

The diagnosis of CVID is frequently difficult due to its diverse clinical manifestations and the lack of universally recognized diagnostic criteria. The latest International Consensus Document (ICON) guidelines define five criteria necessary for diagnosis. (1) Serum IgG levels exceeding two standard deviations below the age‐adjusted reference range, confirmed on a minimum of two occasions separated by more than 3 weeks unless levels are severely diminished (< 100–300 mg/dL, contingent on age); (2) a concurrent reduction in either IgA or IgM; (3) compromised antibody responses to vaccination; (4) age exceeding 4 years; and (5) exclusion of secondary aetiologies of hypogammaglobulinemia [90].

The diagnostic criteria put forward by the European Society for Immunodeficiencies (ESID) vary in numerous significant respects. The ESID definition mandates a reduction in IgA, and the assessment of switched memory B cells (with levels below 70% of the age‐specific reference range) may act as a substitute for vaccination response evaluation. Furthermore, it is essential to establish the lack of significant T‐cell deficiency, and patients must exhibit at least one clinical manifestation, including heightened vulnerability to infections, autoimmune disorders, granulomatous inflammation, unexplained polyclonal lymphoproliferation, or a familial history of antibody deficiency [91]. Tables 1 and Table 2 summarise the most commonly used diagnostic criteria for CVID.

**TABLE 1:** Revised ESID (2014) diagnostic criteria for CVID [138].

Category	Criteria	Details
Clinical manifestations (≥ 1 required)	Increased susceptibility to infection	
	Autoimmune manifestations	
	Granulomatous disease	
	Unexplained polyclonal lymphoproliferation	
	Family history	First‐degree relative with antibody deficiency
Immunoglobulin levels	Marked decrease of IgG and IgA	With or without low IgM; measured ≥ 2 times; < 2 SD below age‐related normal
Immune dysfunction (≥ 1 required)	Poor antibody response to vaccines/absent isohemagglutinins	Absence of protective antibody levels despite vaccination
	Low switched memory B cells	< 70% of age‐related normal values
Exclusion criteria	Secondary causes of hypogammaglobulinemia	Must be ruled out
Age criterion	Diagnosis after age 4 years	Symptoms may present earlier
No profound T‐cell deficiency (all must be met; defined as absence of ≥ 2 of the following)	CD4 T‐cell counts	2–6 years: < 300/μl; 6–12 years: < 250/μl; > 12 years: < 200/μl
	Naïve CD4 T‐cell percentages	2–6 years: < 25%; 6–16 years: < 20%; > 16 years: < 10%
	T‐cell proliferation	Absent

**TABLE 2:** Proposed diagnostic criteria for CVID by Ameratunga et al. [adapted from ref. 139].

Category	Criteria	Details
A. Must meet all major criteria	Hypogammaglobulinemia	IgG < 5 g/L
	No other cause for immune defect	Secondary causes excluded
	Age > 4 years	
B. Sequelae attributable to immune system failure (≥ 1)	Recurrent, severe, or unusual infections	
	Poor response to antibiotics	
	Breakthrough infections despite prophylaxis	
	Infections despite vaccination	e.g., HPV disease
	Bronchiectasis/chronic sinus disease	
	Inflammatory disorders/autoimmunity	
C. Supportive laboratory evidence (≥ 3)	Reduced IgA (< 0.8 g/L) and/or IgM (< 0.4 g/L)	
	Abnormal B cell subsets	Reduced memory B cells and/or ↑CD21^low^ subsets
	IgG3 deficiency	< 0.2 g/L
	Impaired vaccine responses	vs. age‐matched controls
	Transient vaccine responses	vs. age‐matched controls
	Absent isohemagglutinins	If not blood group AB
	Serological autoimmunity	e.g., Coombs test
	Genetic variants predisposing to CVID	e.g., TACI, BAFFR, MSH5
D. Histological markers (not required, but supportive)	Lymphoid interstitial pneumonitis	
	Granulomatous disorder	
	Nodular regenerative hyperplasia of the liver	
	Nodular lymphoid hyperplasia of the gut	
	Absence of plasma cells on gut biopsy	

*Note:* Meeting the criteria in categories ABC or ABD suggests a probable diagnosis of CVID. Patients who satisfy criteria ABC and ABD should receive IVIG/SCIG treatment. Patients who satisfy only criterion A, or criteria AB, AC, or AD without criterion B, are classified as potential CVID. Certain patients may require treatment with IVIG or SCIG. Patients exhibiting IgG levels over 5 g/L, without fulfiling any additional criteria, are classified as having hypogammaglobulinemia of undetermined significance. The diagnostic criteria must be employed in sequence, as none are individually specific.

### 5.2 CVID Enteropathy

The definition of CVID‐associated enteropathy is complex and largely based on the exclusion of other aetiologies. Early descriptions characterised it as villous atrophy with intraepithelial mucus and lymphocytic infiltration of the lamina propria, unresponsive to gluten withdrawal [70]. Over time, a broader spectrum of histological lesions—including intraepithelial lymphocytosis, intestinal atrophy, follicular lymphoid hyperplasia, and acute or chronic duodenitis—came to be recognized as characteristic [31]. Subsequently, the condition was defined more pragmatically as an enteropathy of unclear origin associated with chronic diarrhoea in the absence of infection, autoimmunity, or gluten sensitivity [31, 71]. More recent refinements have incorporated clinical criteria, such as chronic diarrhoea or unexplained weight loss exceeding 5% of body weight within 6 months, accompanied by changes in parameters including BMI, serum albumin, serum protein, or stool frequency [72]. Histological features supporting the exclusion of infectious causes have also been emphasised [73, 74, 75]. Table 3 summarises the criteria for CVID enteropathy.

**TABLE 3:** Proposed criteria and features of CVID‐associated enteropathy.

Category	Criteria/features	Details/references
Definition	Exclusion‐based diagnosis	Absence of infection, autoimmunity, or gluten sensitivity [31, 71]
Early descriptions	Villous atrophy	With intraepithelial mucus and lymphocytic infiltration of the lamina propria; unresponsive to gluten withdrawal [70]
Histological spectrum	Intraepithelial lymphocytosis	[31]
	Intestinal atrophy	[31]
	Follicular lymphoid hyperplasia	[31]
	Acute or chronic duodenitis	[31]
Clinical definition (pragmatic)	Enteropathy of unclear origin	Associated with chronic diarrhoea, absence of infection/autoimmunity/gluten sensitivity [31, 71]
Refined clinical criteria	Chronic diarrhoea or unexplained weight loss	> 5% of body weight within 6 months [72]
	Changes in nutritional/clinical parameters	BMI, serum albumin, serum protein, stool frequency [72]
Supporting features for exclusion of infectious causes	Clinical and histological findings	Raised faecal calprotectin, signs of malabsorption, impaired quality of life, altered microbial diversity, graft versus host disease‐like histopathological changes [73, 74, 75]

### 5.3 Chronic Norovirus Enteritis

Chronic norovirus enteritis ought to be investigated in immunocompromised individuals with persistent or recurrent diarrhoea, weight loss, and malabsorption symptoms [102]. Standard microbiological tests typically produce negative findings, and confirmation requires the repeated detection of norovirus RNA in faeces with RT‐PCR, the final diagnostic standard [103, 104]. Histopathological indicators from intestinal biopsies, such as villous blunting, crypt hyperplasia, and intraepithelial lymphocytosis, may support the diagnosis; however, they are nonspecific and often overlap with other forms of immune‐mediated enteropathy [89, 92, 93]. Consequently, a synthesis of consistent clinical features, exclusion of other aetiologies, and sustained stool PCR positivity validates the diagnosis.

## 6 Therapeutic Possibilities

In patients on immunosuppressive therapy, reducing these medications may help resolve norovirus infections, but this is not possible for patients with PID, and there are no approved vaccines or therapeutic agents [105, 106, 107]. Ribavirin and nitazoxanide are off‐label norovirus treatments [18, 108, 109]. A nucleoside analogue, ribavirin, treats persistent hepatitis C infections. Broad‐spectrum antiparasitic nitazoxanide has been examined as an antiviral, which inhibits norovirus replicon replication [110]. Interferon 2‐alpha may also reduce norovirus shedding in pigs [105, 111].

The therapeutic efficacy of ribavirin in CVID‐associated enteropathy complicated by norovirus infection has been reported only in a limited number of cases [18, 112, 113, 114]. The mean duration of infection was approximately 2.5 years, although in some patients it persisted for more than 6 years [112]. Given the impaired mucosal absorption of ribavirin in CVID enteropathy, monitoring of drug levels is recommended [19]. In selected cases, trough concentrations were measured, and the lowest effective therapeutic level was determined to be 1000 ng/mL [18]. Despite this, infection clearance was achieved in only two of seven patients after 1 year of treatment, while therapeutic failure has also been observed in individuals receiving doses exceeding this threshold [112].

It is believed that the antiviral action of nitazoxanide works either by boosting the host's innate immune response or by blocking the maturation of viral proteins [110, 115, 116]. In norovirus infection, initial findings were promising; however, following investigations regarding chronic infection have produced inconsistent effects [117, 118, 119, 120]. One potential reason for treatment failure is the biological heterogeneity across norovirus genotypes, encompassing disparities in cellular entry mechanisms and sensitivity to host interferon pathways [45, 121, 122].

IVIG and SCIG treat numerous infections in PID patients. To ensure a wide range of antibody specificities against various illnesses, these immunoglobulin preparations are made from plasma from multiple (from 1000 up to 100,000) donors [123, 124]. In persistent norovirus infections, immunoglobulins are given directly to the gut via nasoduodenal tubes (i.e.,: OHIG) [125, 126].

The above treatments have been empirically tested with varying success rates, resulting in quick cures for some individuals but not others [18, 19, 31, 79, 105, 127]. Most of these investigations typically failed to assess virus genotypes, shedding patterns, and clearance processes throughout therapy, confounding the assessment of treatment outcome variability and therapeutic improvements. However, two new norovirus cell culture models—one using B cells and the other using human intestinal enteroids—may help improve chronic norovirus infection treatment [57, 128, 129].

## 7 Our Experience With CVID Enteropathy Associated With Chronic Norovirus Infection

Regarding the past medical history of the 70‐year‐old female patient, she underwent an appendectomy, left‐sided breast surgery due to a benign tumour, and a laparoscopic cholecystectomy.

Since the 2010s, she complained several times of cough, haemoptysis, brownish sputum, high‐grade fever (40°C), and chills and was diagnosed repeatedly with respiratory tract infections, mainly bronchitis and pleuro‐pneumonias. Sputum cultures occasionally indicated *Hemophilus influenzae* and *Staphylococcus aureus* (but no *mycobacteria*), with markedly increased inflammatory markers (ESR over 100 mm/h, CRP 350 mg/L). Repeated chest CT scans revealed no evidence of malignancy but demonstrated extensive pneumonias and enlarged lymph nodes in the mediastinal and subcarinal hilar regions. The lymphadenopathy was considered secondary to pneumonia. Bronchoscopy indicated purulent secretions. Cytology from a peribronchial puncture contained erythrocytes and inflammatory cells. She was treated again and again with IV and oral antibiotics, resulting in significant clinical and radiological regressions.

In 2021, despite displaying chronic watery diarrhoea for 5–6 years, she was treated with ciprofloxacin for a *Salmonella* GI tract infection (other bacteria including *Clostridium difficile* were not detected). The colonoscopy was unremarkable. A food allergy panel and coeliac serology performed were also negative.

**FIGURE 2:**
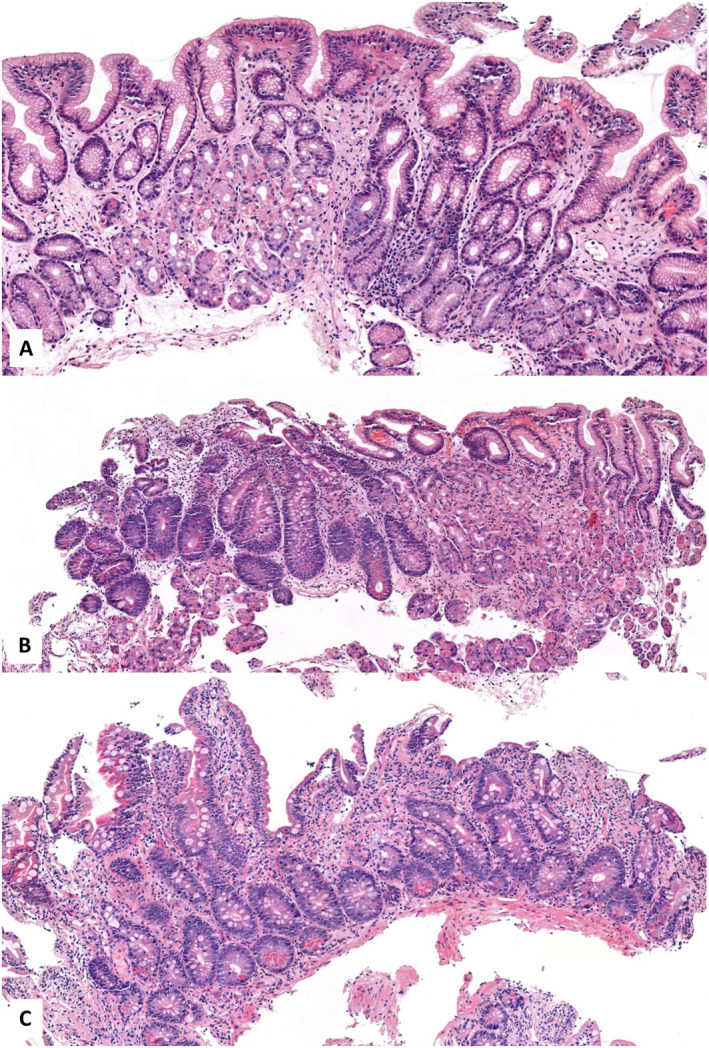
Representative histology illustrations. (A) Evidence of chronic atrophic gastritis with full intestinal metaplasia is observed in the antrum and (B) the corpus. (C) Partial villous atrophy with intraepithelial lymphocytosis in the postbulbar duodenum. (Staining with haematoxylin and eosin. Magnification: A: 15.8x; B: 9.2x; C: 11.7x.).

In May 2023, gastroscopy revealed atrophic gastritis with a histology of partial villous atrophy accompanied by intraepithelial lymphocytosis in the postbulbar duodenum, as well as chronic atrophic gastritis featuring complete intestinal metaplasia in the gastric biopsies. No *Helicobacter pylori* strains were detected (Figure 2). Though coeliac disease screening was also negative, a gluten‐free diet was introduced. Repeated colonoscopy revealed no macroscopic abnormalities; histology was non‐specific. The stool test for *Giardia lamblia* and *Clostridium difficile* was negative.

Endocrinological evaluation in November 2023 excluded a neuroendocrine tumour such as VIPoma. Chromogranin A was moderately elevated, likely attributable to atrophic gastritis, while 5‐HIAA was at the upper normal limit. Somatostatin receptor SPECT/CT did not confirm a neuroendocrine tumour.

A hereditary angioedema panel performed in November 2023 was negative, with normal complement parameters and C1‐inhibitor activity.

MR enterography in January 2024 revealed no space‐occupying lesions, strictures, or significant abnormalities in the gastrointestinal tract, although duodenal folds appeared slightly thinned.

In February 2024, the patient was evaluated at our Clinical Department for immunological consultation. She reported a history of chronic diarrhoea lasting for 7–8 years, accompanied by fatigue and unintentional weight loss of over 30 kg for the past 3–4 years, with a current body weight of 37 kg (BMI: 15.6 kg/m^2^).

More recently, she noticed cold‐induced blanching and transient numbness of her fingers, suggestive of Raynaud's phenomenon.

Further laboratory testing revealed markedly reduced serum immunoglobulin (IgG, IgA, IgM) levels. Flow cytometry of peripheral blood lymphocytes showed significantly decreased proportions of CD27^+^/IgD^−^ switched memory B cells, as well as non‐switched memory B cells, while CD27^‐^/IgD^+^ naïve B cells were disproportionately increased.

Coeliac disease HLA typing was negative for DQ2 and DQ8 alleles.

Multiplex PCR stool analysis and melting point analysis (BioFire FilmArray GI panel) yielded positive results for norovirus GII. Other tested bacterial, viral, and parasitic pathogens—including *Salmonella*, *Shigella*, *Yersinia enterocolitica*, *Campylobacter*, *Clostridium difficile*, *Vibrio cholerae*, pathogenic *E. coli* strains (EAEC, EPEC, ETEC, EIEC, STEC), Adenovirus F, Astrovirus, Rotavirus A, Sapovirus (genotypes I, II, IV, and V), *Cryptosporidium*, *Cyclospora cayetanensis*, *Entamoeba histolytica*, and *Giardia lamblia*—were not detected. The BioFire FilmArray result was also confirmed by the Viasure Multiplex Norovirus GI + GII Real Time PCR Detection Kit (the cycle threshold/Ct/value was 26).

Based on these findings, a diagnosis of CVID was established. The patient was considered to have CVID‐associated sprue‐like chronic enteropathy, norovirus‐associated chronic diarrhoea, malabsorption syndrome, and malnutrition. At this stage, there was no evidence of systemic autoimmune disease.

In March 2024, the patient was hospitalised in our immunology department. Given the presence of severe pan‐hypogammaglobulinemia affecting all three immunoglobulin classes, intravenous immunoglobulin (Intratect) substitution was initiated, consisting of two infusions of 5 g each. In the context of pronounced malnutrition, the patient also received parenteral nutritional support, including glucose, a balanced mixture of amino acids and fatty acids, as well as intravenous multivitamin supplementation. Electrolyte replacement therapy was introduced to correct hypocalcemia, hypokalemia, and hypomagnesaemia. Subsequently, enteral nutritional support was initiated with Modulen IBD powdered formula and Nutridrink Max oral formula, both of which were well tolerated.

The patient's IgG trough concentration was initially extremely low (< 0.7 g/L), but with the administration of 20–30 g IVIG every four weeks, the IgG level increased gradually to the expected trough level of 5–6 g/L.

However, repeated stool RT‐PCR tests [BioFire FilmArray and Viasure Multiplex Norovirus GI + GII Real Time PCR Detection Kit (Ct was 29)] confirmed the persistent positive result for norovirus GII. Based on previous case reports [115, 120], with some modifications, in January 2025, during close immunological monitoring, we initiated oral human immunoglobulin (OHIG) supplementation via nasogastric tube. The patient received 15 g of Intratect immunoglobulin on two occasions, 5 days apart, each administered in the morning on an empty stomach. The preparation used was a 50 g/L Intratect solution (supplied in 100 mL vials) with the following approximate IgG subclass distribution: 57% IgG1, 37% IgG2, 3% IgG3, and 3% IgG4. The maximum IgA content of the solution is 900 μg/mL. For each administration, a total of 300 mL of solution was prepared and delivered in 30 mL aliquots every 20 min through the nasogastric tube.

In February 2025, repeated stool RT‐PCR again detected norovirus; however, the patient reported a near‐complete resolution of diarrhoea and had gained 2 kg in body weight. At that time, her IgG trough concentration had risen to 5.2 g/L. In March 2025, the patient underwent a second cycle of OHIG treatment as described above, receiving 15 g on two occasions, five days apart.

Subsequent stool RT‐PCR testing in April and June 2025 was negative for norovirus. The patient's body weight continued to increase, reaching 52 kg, and IgG trough levels stabilised between 4.9 and 5.4 g/L under ongoing monthly 30 g IVIG therapy. A follow‐up colonoscopy performed in April 2025 confirmed completely normal mucosal appearance both macroscopically and histologically.

## 8 Discussion

Chronic norovirus infection has become a significant contributor to the development of enteropathy linked with CVID. More than 20% of CVID patients have gastrointestinal problems [8, 9, 32]. Norovirus has been linked to both frequent infections and chronic mucosal inflammation and villous atrophy [17, 18, 19, 69]. Moreover, *Clostridium difficile* toxins can disturb intestinal homoeostasis, increasing vulnerability to norovirus co‐infections [130, 131].

When diagnosing norovirus infection, it is important to note that false positive results have been observed with the BioFire FilmArray method [132], with the reported false positive rate reaching up to 36% for norovirus GI. However, BioFire FilmArray is a CE‐IVD [European Union's In Vitro Diagnostic Regulation (IVDR 2017/746)] certified test, so ‐in general‐there is no need for subsequent verification of the results. If the manufacturer finds an error in the test, it will notify users. In the case of norovirus, errors occurred on several occasions, in which case the Microbiology Laboratory of the Institute of Laboratory Medicine at Semmelweis University used an immunochromatographic antigen detection test to confirm the positive results. The stool norovirus tests conducted on our patient during the relevant period did not exhibit any errors. Nevertheless, the patient's stool sample was further examined at the National Centre for Public Health and Pharmacy, where the BioFire results were validated using another PCR method.

Persistent norovirus replication has been recognized as a principal etiological factor of CVID enteropathy, a serious non‐infectious consequence marked by chronic diarrhoea, malabsorption, weight loss, and diminished quality of life [31, 71, 78].

The pathophysiological relationship between CVID and norovirus persistence is complex. Impaired humoural immunity, especially IgA deficiency [13], and abnormal B‐cell maturation [68, 69], impede viral clearance. As a result, a long‐lasting infection keeps damaging epithelial cells through uncontrolled CD8+ T‐cell responses and immune‐mediated tissue damage [69]. Significantly, viral clearance has been linked to both clinical and histological enhancement, which emphasises the aetiology of norovirus in this distinct enteropathy [18, 19].

Nonetheless, therapeutic alternatives continue to be inadequate. Standard immunoglobulin replacement therapy (IVIG/SCIG) offers inadequate protection against GI infections [12], and OHIG (oral/via nasogastric or nasoduodenal tube/administration of human immunoglobulin) has yielded variable results [125, 126]. The diversity probably comes from changes in the viral genotype, host factors, and the range of neutralising antibodies in preparations made from donors [18, 19, 31, 79, 105, 127]. Likewise, off‐label antivirals like ribavirin and nitazoxanide have demonstrated sporadic and unexpected efficacy [18, 108, 109]. These limitations underscore the absence of standardized, evidence‐based methodologies, as the majority of data is sourced from case reports or small series, with no randomized clinical trials available.

The future appears promising. Sophisticated norovirus culture methods, such as human intestine enteroids [128, 129], may enhance our understanding of viral persistence and treatment efficacy. Simultaneously, it is essential to delineate the immunological correlates of viral clearance and to investigate targeted antiviral or vaccination methods. Until such advancements occur, the management of chronic norovirus‐associated CVID enteropathy must remain personalised, incorporating supportive care, meticulous nutritional monitoring, and targeted therapeutic interventions. Recognising chronic norovirus infection as a primary etiological factor in CVID enteropathy signifies a paradigm change, underscoring the necessity for systematic investigations to enhance diagnostic, therapeutic, and preventive methods in this susceptible patient cohort.

What factors contributed to the success of the OHIG treatment we implemented? In our patient's case, we could not evaluate the Intratect preparation utilised to ascertain the norovirus‐specific neutralising antibody titre. Nevertheless, the patient's clear clinical improvement confirms that OHIG treatment was ‐at least partially‐effective in neutralising the virus. Throughout the treatment, our patient was supplied a cumulative total of 60 g (15 g per dosage) of OHIG on four separate occasions, exceeding the quantities reported in other case studies [123, 133], where patients got roughly 4 g per day. It is believed that 3 g of IgA and somewhat less IgG are secreted daily in the digestive tract [134]. In our case, 270 mg of remnant IgA per treatment was used, resulting in a cumulative total of 1080 mg of IgA. The residual IgA from the Intratect preparation may have contributed, although the IgG utilised may have mitigated the absence of mucosal IgA.

It can be inferred that OHIG treatment did not accomplish total viral eradication; nonetheless, it successfully diminished the quantity of infectious virus in the intestine momentarily, enabling the patient's immune system to eliminate the residual infection. The reported failure of OHIG treatment in certain instances may be attributed to norovirus mutation or the presence of a viral strain against which the donors who participated in the immunoglobulin preparation did not generate neutralising antibodies. Consequently, it would be prudent to standardise immunoglobulin preparations according to the concentration of norovirus‐specific neutralising antibodies or, alternatively, utilise convalescent plasma, akin to therapies for SARS‐CoV‐2 [105].

There is also evidence that some of the immunoglobulin administered orally avoids degradation in the stomach and reaches the small intestine [135].

For our patient, we administered OHIG therapy alongside IVIG treatment. While lacking precise findings, one cannot dismiss the putative antiviral function of the idiotype network modulated by immunoglobulin therapy in the mucosa [136, 137].

## 9 Future Perspectives

Existing evidence for the application of oral immunoglobulin for norovirus infection treatment is scarce, predominantly derived from case studies and limited trials. These studies may exhibit publication bias, thus rendering the generalisability of the findings unclear. A notable disadvantage is the absence of high‐quality, randomized clinical trials, essential for validating the treatment's efficacy and establishing the best dose regimen.

The mechanism of action of immunoglobulin is intricate; it may exhibit both anti‐inflammatory and pro‐inflammatory characteristics, and the localised intestine effects of oral treatment remain inadequately comprehended. This necessitates additional inquiry to enhance comprehension of the pharmacodynamic and immunomodulatory mechanisms.

Additional methodological issues further obfuscate the interpretation of the data. The mode of delivery, such as nasoduodenal or nasogastric, can differ markedly across studies, thereby influencing efficacy and leading to result discrepancies. This constrains the extensive extrapolation of the data.

The existing data are encouraging; yet, the evidential quality is minimal. Future well‐structured, randomized, controlled trials are essential to elucidate the conditions, dosage, and delivery route for the effective use of oral immunoglobulin in treating norovirus infection.

## Author Contributions

Conceptualisation: G.M. and F.S. Writing – original draft preparation: G.M. and F.S. Writing – review and editing: G.M. and F.S. Visualisation: F.S. Supervision: G.M. and F.S. All authors have read and agreed to the published version of the manuscript.

## Funding

The authors have nothing to report.

## Ethics Statement

The Institutional Review Board was contacted, and the authors were assured that, as this is a review article and no research was being conducted, this manuscript was not under their jurisdiction.

## Consent

The patient provided written consent for the publication of her clinical case in this review article that describes her disease's symptoms but prevent her identification.

## Conflicts of Interest

The authors declare no conflicts of interest.

## Permission to Reproduce Material From Other Sources

The authors have nothing to report.

## Data Availability

The original contributions presented in this study are included in the article. Further enquiries can be directed to the corresponding authors.
